# Habitat environments impacted the gut microbiome of long-distance migratory swan geese but central species conserved

**DOI:** 10.1038/s41598-018-31731-9

**Published:** 2018-09-06

**Authors:** Yueni Wu, Yuzhan Yang, Lei Cao, Huaqun Yin, Meiying Xu, Zhujun Wang, Yangying Liu, Xin Wang, Ye Deng

**Affiliations:** 1 CAS Key Laboratory for Environmental Biotechnology, Research Center for Eco-Environmental Sciences, Chinese Academy of Sciences, Beijing, China; 2 College of Resources and Environment, University of Chinese Academy of Sciences, Beijing, China; 3 State Key Laboratory of Urban and Regional Ecology, Research Center for Eco-Environmental Sciences, Chinese Academy of Sciences, Beijing, China; 4 School of Minerals Processing and Bioengineering, Central South University, Changsha, China; 5 State Key Laboratory of Applied Microbiology Southern China, Guangdong Institute of Microbiology, Guangzhou, China

## Abstract

The gut microbime plays an important role in the health of wild animals. This microbial community could be altered by habitat pollution and other human activities that threaten the host organisms. Here, we satellite-tracked a flock of swan geese (*Anser cygnoides*) migrating from their breeding area (Khukh Lake, Mongolia), with low levels of human activity, to their wintering area (Poyang Lake, China) which has been heavily impacted by human activities. Twenty fecal samples were collected from each site. High-throughput sequencing of 16S and ITS was employed to explore bacterial and fungal composition and diversity of their gut microbiome. Although general composition, alpha-diversity, functional prediction, and the central taxa in the phylogenetic networks showed some similarities between the two habitats, significant divergences were detected in terms of beta-diversity, species abundances, and interaction network topologies. In addition, disease-related and xenobiotic biodegradation pathways, and pathogenic bacteria were significantly increased in bacterial communities from samples at Poyang Lake. Our results reveal that the gut microbiome of swan geese, while somewhat altered after long-distance migration, still maintained a core group of species. We also show that habitat environmental stress could impact these gut microbial communities, suggesting that habitat pollution could indirectly threaten wild animals by altering their gut microbiome.

## Introduction

**Figure 1:**
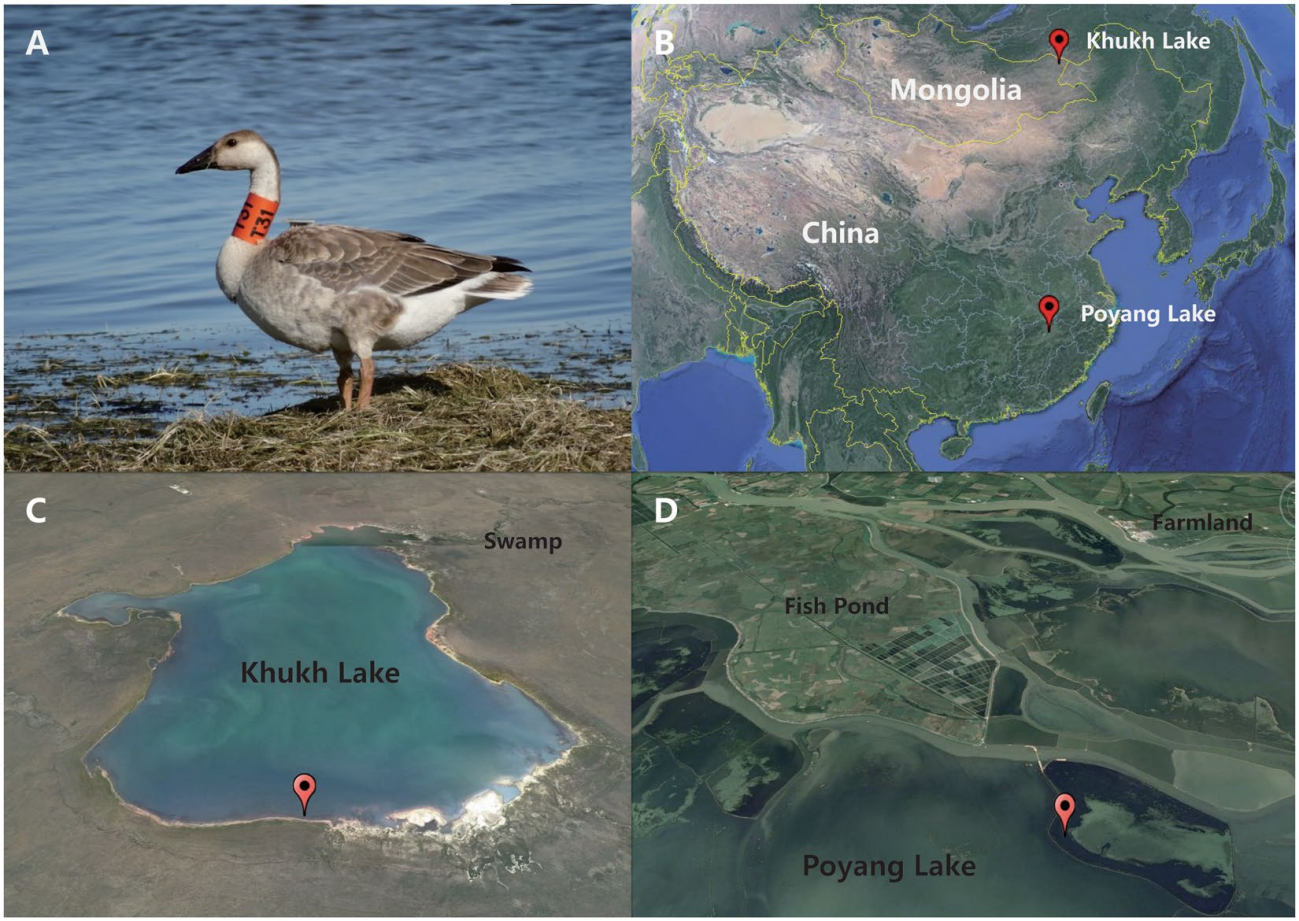
Basic information of swan geese and their habitat (**A**) Physical characteristic of swan geese. (**B**) Migration route of swan geese. (**C**) Surrounding environment of breeding area. (**D**) Surrounding environment of wintering area. All the maps (Fig. 1B,C and D) were generated by Google Earth. Map data ©2018 Google.

The swan goose (*Anser cygnoides*), a wetland-dependent herbivore waterbird, is a representative species of wild Anseriformes of Anatidae (Fig. 1A). These wild geese migrate thousands of miles every year between their breeding and wintering areas. A majority breed in central and eastern Mongolia and adjacent regions of China and Russia, while the main wintering area for this species is the Middle and Lower Yangtze River floodplain wetlands in eastern China^1^. Swan geese mainly subsist on the young stems of submerged macrophytes, particularly those of *Vallisneria spiralis*, in both their wintering and breeding areas^2^. This narrow dietary range makes this species highly sensitive to environmental changes^1^. Due to habitat loss, excessive hunting, and egg collection, the population of the geese has dramatically declined over the past couple decades. The swan goose was uplisted on the IUCN (International Union for Conservation of Nature) Red List from Near Threatened to Vulnerable in 1992 and further elevated to Endangered in 2000^3^. Based on combined counts during the non-breeding season in East Asia, the total number of swan geese was 56,000–98,000^4^. While a great deal of effort has been invested in the study of habitat changes and migration routes for its conservation^1^, there has been less concern about how habitat impacts affect the symbiotic microbiome of swan geese.

The gut microbiome is known to play important roles in food digestion, metabolism regulation, and the immune protection of animals^5,6^. During the last decade there have been rapid developments in the investigation of community composition and structure of vertebrate gut microbiota and interactions with their hosts^7,8^. Because of their distinct morphological characteristics and high-energy requirements^9^, the avian gut microbiome is significantly different from those of other animals^10–12^. It should also be noted that these studies have mainly focused on either economically important avians, such as chickens and turkeys, or rare wild species, such as hoatzins^13^, kakapo^14^, penguins^15^, vultures^16^, *Procellariiformes* seabirds^17^ and hooded cranes^18^. Little is currently known about the gut microbiota of herbivorous geese. Wild *Anseriformes* of *Anatidae* are exceptional herbivores in bird lineages, as most are exclusively herbivorous, feeding on leaves, tubers, seeds, or rhizomes of plants^19^. Swan geese require high-protein foods, such as young tubers of *Vallisneria natans*, to obtain sufficient energy^1^.

Swan geese migrate annually between breeding and wintering areas in response to seasonal fluctuation in temperature and food availability^20^. Therefore, their gut microbial community plays an important role in the geographic spread of various zoonotic agents, because it is one of the major ways to transport pathogenic bacteria^21^. Because of their food resources, overwintering and reproduction wetland refuges play very important roles in the migration of birds with a narrow dietary range. As the living areas for most wild animals, wild habitats have huge impacts on the health of all residents, including their behavior, foraging, growth, and breeding^22,23^. Human activity and environmental pollution have become serious threats to these areas^24^. The host species is considered to be one important factor in shaping the gut microbial structures^25^, and dietary nutrients as a second important factor^26^. The assemblage patterns of birds’ gut microbiome were also driven by nutrients and host species^25,27^, but the impacts from the habitat environment have not been cautiously studied.

Our previous study proved that both host species and dietary nutrients are potential drivers of geese gut microbiome assemblages, while among different goose species the functions of gut microbiome could be similar in their wintering areas^28^. However, how the distinctly different post-migration habitats affect gut microbiomes remained unknown. In this study, we tracked a flock of swan geese by satellite as they migrated from their breeding to wintering area (1800 km apart) and studied how their gut microbiome changed. The breeding and wintering area of swan geese display different levels of environmental stress. The breeding area, Khukh Lake, is minimally impacted by human activities while their wintering area Poyang Lake sees high level of human activities including fisheries aquaculture. Water quality of Poyang Lake has declined in recent years, particularly due to input from agricultural drainage water with high total nitrogen and phosphorus concentrations caused by increasingly intensive use of chemical fertilizers by local farmers^29^. Thus, we hypothesized, (i) the core group of gut microorganisms are similar due to the same host species and similar diet; while (ii) both bacterial and fungal communities could be altered after long distance migration.

## Results

### Breeding and wintering habitats and gut microbiome sequence information

In north, swan goose fecal samples were collected at Khukh Lake, Mongolia. This wildlife refuge is located in eastern Mongolia, which has low levels of human activities (Fig. 1C). Following satellite tracking of the flock (Fig. 1B), fecal samples from the same group of swan geese were collected in Poyang Lake, South China. The Poyang Lake has high level of human activities, including fisheries aquaculture and cultivation (Fig. 1D). Metagenomic DNA was extracted from a total of 40 samples and 6,076,016 16S high quality paired sequences were obtained. Thereafter, all high quality sequences were assigned to 23,978 OTUs at the level of 97% similarity. Furthermore, sequencing of the fungal ITS (Internal transcribed spacer) generated a total of 1,576,427 sequences for fungi community analysis which, after basic processing and quality filtering, were assigned to 1,741 OTUs at the level of 97% similarity. Rarefaction curves of all 40 samples, for both 16S and ITS, tended to be saturated (Fig. S1) indicating the sequencing depths for these samples were appropriate.

### Composition of the swan goose gut microbiota

**Figure 2:**
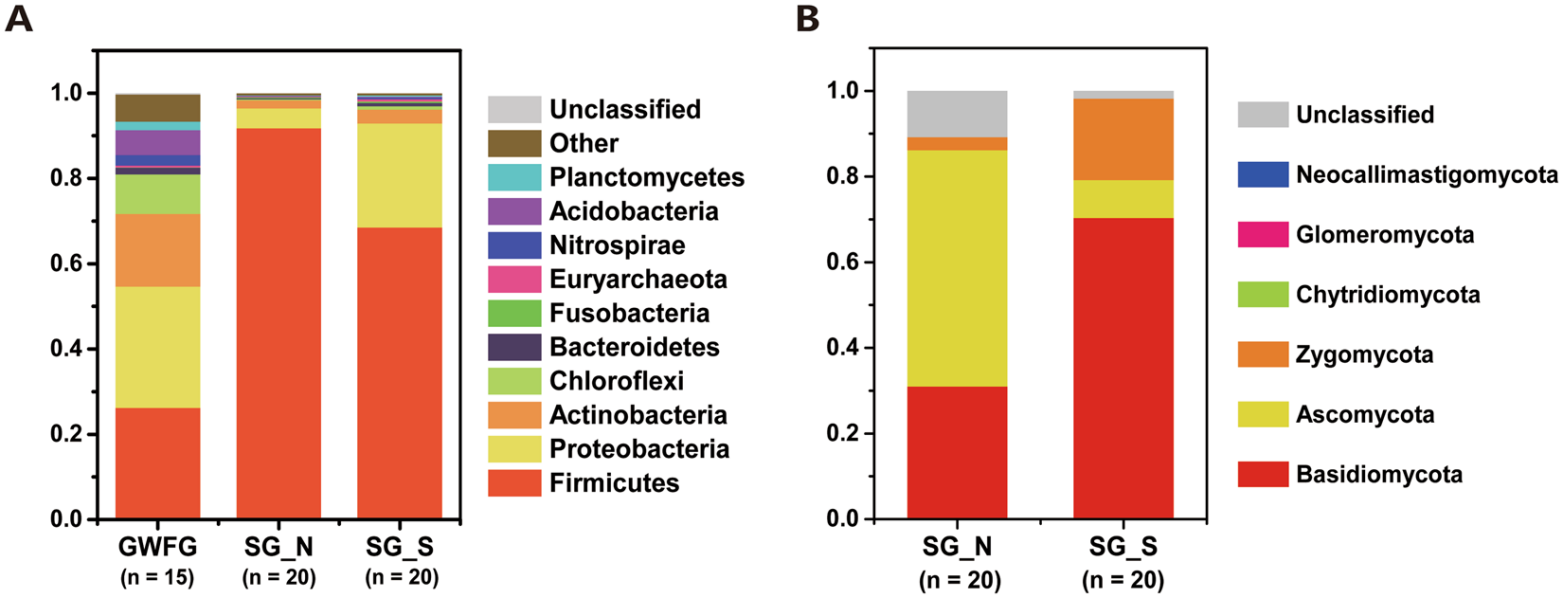
Phylum-level gut microbiome composition of herbivorous geese. (**A**) 16S phyla in all samples. (**B**) ITS phyla in all samples. Samples are grouped according to sampling location and species.

The swan goose 16S rRNA sequences could be assigned to 37 bacterial phyla (Fig. 2A). There was a relatively low abundance of unclassified Bacteria (0.03%). The top three dominant phyla, accounting for 98% of the relative abundance, were *Firmicutes* (83.55%), *Proteobacteria* (11.63%), and *Actinobacteria* (2.00%). At lower taxonomic levels, 1,486 genera were detected across all samples. More than 90% of the sequences belonged to 16 genera, with the dominant genera being *Clostridiaceae-*SMB53 (23.21%), *Turicibacter* (17.75%), and *Clostridium* (8.39%). At the phylum level, samples from both sites were dominated by *Firmicutes* (SG_N 91.78%, SG_S 68.52%), no significant difference (T-test *P* = 0.119) between the two habitats (Fig. S2). At the genus level, *Clostridiaceae-*SMB53 (23.21%) was dominant, which, again, was not significantly different (T-test, *P* = 0.07) between the study sites, SG_N (24.77%) and SG_S (21.58%). The differences between each site derived from discrepancies in abundance at both phylum and genus levels. At phylum level (Fig. 2A), a large proportion of *Proteobacteria* were found in Poyang Lake samples of both swan geese and the greater white-fronted geese (GWFG)^28^ (SG_S 24.40%, GWFG 28.45%), including *Vibrio, Shewanella, Pseudoalteromonas*, (T-test, *P* < 0.001) and other *Gamma-proteobacteria* (Figs S3, S4C). However, the proportion of *Proteobacteria* was relatively small in swan goose samples from Khukh Lake (SG_N 4.64%). Relatively significant difference was shown in these two habitats (T-test *P* = 0.002) (Fig. S2). At the genus level, several genera were uniquely enriched between SG_N and SG_S, including *Clostridium* (SG_N 3.35%, SG_S 16.32%, *P* = 0.002), *Turicibacter* (SG_N 17.69%, SG_S 0.39%, *P* < 0.001), *Lactobacillus* (SG_N 1.58%, SG_S 11.52%, *P* = 0.009), and *Solibacillus* (SG_N 8.17%, SG_S 1.61%, *P* = 0.001) (Figs S4B, S4C).

Swan goose gut microbiome ITS sequences could be assigned to 6 phyla (Fig. 2B), with a relatively high abundance of unclassified Fungi (7.16%). The dominant phyla were *Basidiomycota* (48.04%) and *Ascomycota* (30.25%). At lower taxonomic levels, 287 genera were detected across all samples. More than 90% of the sequences belonged to 11 genera, with the dominant genera being *Cryptococcus* (39.20%), *Sporobolomyces* (29.57%), and *Pilaira* (26.58%). A large proportion of unclassified fungi were found in SG_N (11.54%), while the proportion was relatively smaller in SG_S (3.71%). The dominant phylum of SG_N was *Ascomycota* (SG_N 55.51%, SG_S 10.37%, *P* < 0.001), while that of SG_S was *Basidiomycota* (SG_N 30.01%, SG_S 62.22%, *P* < 0.001), and both of these phyla were significantly different between the two sampling areas.

### Variations in the diversities of swan geese’s microbiome between breeding and wintering area

**Figure 3:**
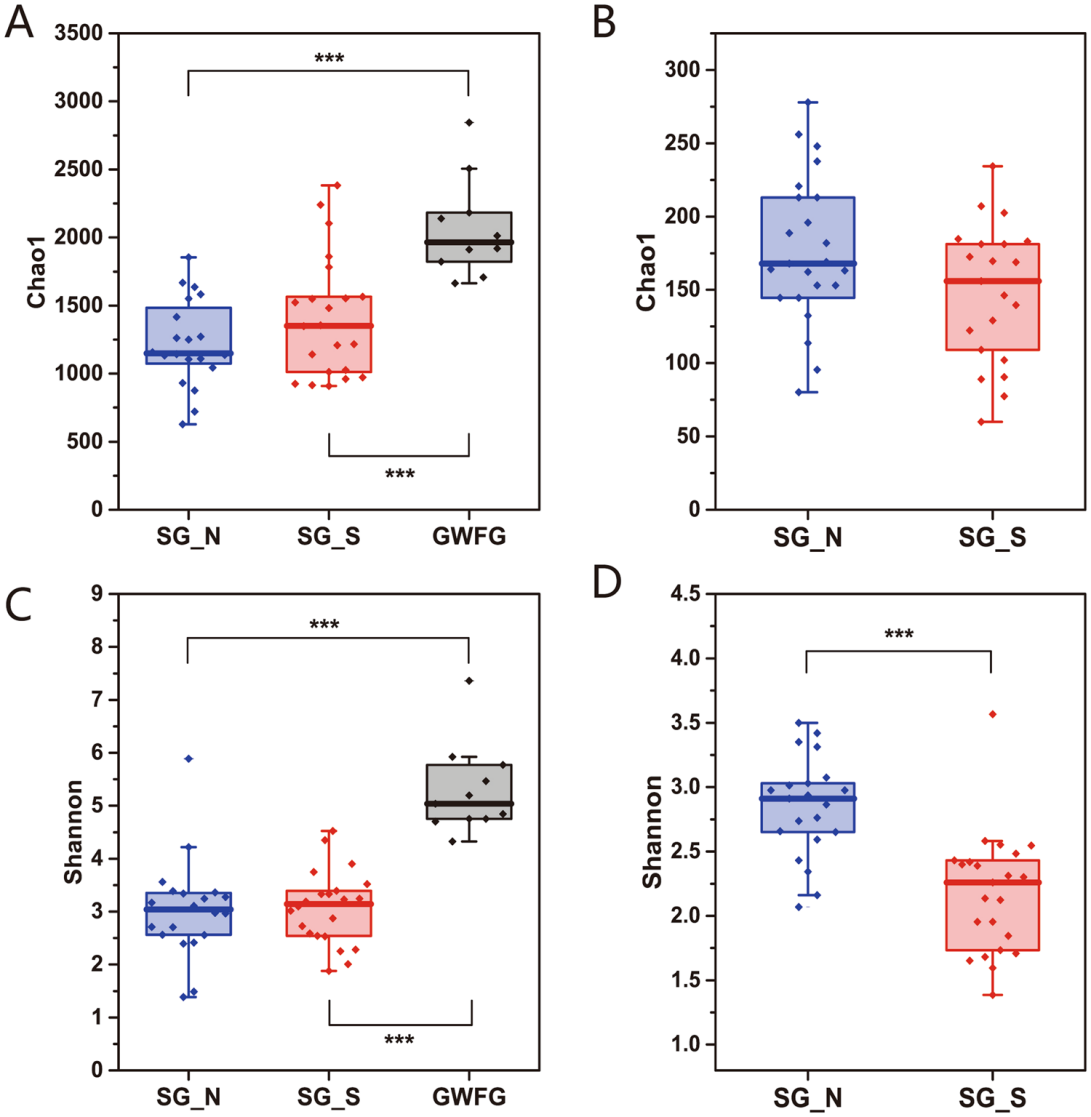
Variations in alpha diversity of herbivorous geese gut microbiome. (**A**) Comparison of 16S Chao1 value between 2 geese species in 2 lakes. (**B**) Comparison of 16S Shannon index between 2 geese species in 2 lakes. (**C**) Comparison of ITS Chao1 value of swan geese between 2 lakes. (**D**) Comparisons of ITS Shannon index of swan geese between 2 lakes. ***P < 0.01.

In order to distinguish the variations in gut microbiomes of swan geese between these two geographically isolated locations, multiple diversity indexes were utilized. Alpha diversity, represented by Shannon index, and the richness, estimated by Chao1 value, both showed that alpha-diversity between the two sites was unchanged (T-test, Shannon *P* = 0.812, Chao1 Value *P* = 0.128) (Fig. 3A,B). Compared with the greater white-fronted geese samples from Poyang Lake^28^ (T-test, Shannon, Chao1 Value both *P* < 0.001), samples from the same species were much more similar (Fig. 3A,B). The fungal Chao1 value showed that the richness between two sites, like bacteria, was also almost unchanged (T-test, *P* = 0.830), however the Shannon index showed a significant difference between two sites (T-test, *P* < 0.001) (Fig. 3C,D).

**Figure 4:**
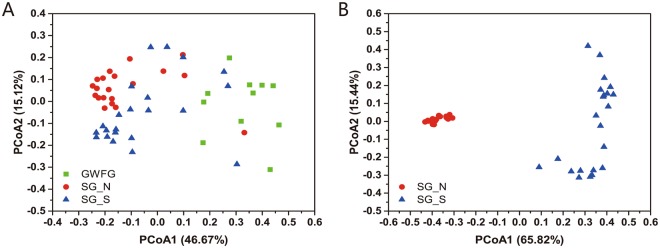
Differential gut microbial communities across all samples. (**A**) Principal coordinates analysis plot of 16S weighted UniFrac distances for three geese species sampled from two lakes. (**B**) Principal coordinates analysis plot of ITS weighted UniFrac distances for swan geese sampled from two lakes. Each point represents the gut microbiome community of an individual geese.

Although most alpha diversity indexes were unchanged, the beta diversity measurements between two sites showed divergence between the two sites. PCoA (Principal coordinate analysis) revealed that, when compared with the greater white-fronted geese^28^, samples from the same species (swan geese) tended to be more similar (Fig. 4A). Dissimilarity tests of gut community structure confirmed this differentiation between SG_S and GWFG, as well as SG_N and GWFG, while the structures of gut microbiome (both bacterial and fungal) between SG_N and SG_S were also different (Table 1). Compared to the bacterial community (ANOSIM, *P* = 0.034; PERMANOVA, *P* = 0.016), the fungal community showed a greater difference (ANOSIM, PERMANOVA, *P* < 0.001) (Fig. 4, Table 2). The PCoA plot also revealed greater inter-individual variation among the SG_S samples than those from SG_N, indicating the gut microbiome varied widely between individual geese in wintering area (Fig. 4).

**Table 1:** Dissimilarity tests of herbivorous geese fecal microbial communities using ANOSIM and PERMANOVA based on Bray-Curtis distance.

Dissimilarity tests	ANOSIM		PERMANOVA	
	R	Significance	R^2^	Significance
SG_N-SG_S(16S)	0.07	0.034*	0.057	0.016*
SG_S-GWFG(16S)	0.414	0.001***	0.162	0.001***
SG_N-GWFG(16S)	0.482	0.001***	0.196	0.001***
SG_N-SG_S(ITS)	0.491	0.001***	0.308	0.001***

*Difference is significant at 0.05 level; **Difference is significant at 0.01 level; ***Difference is significant at 0.001 level.

**Table 2:** Basic information of pMENs.

Network Indexes	Total nodes	Total links	R square of power-law	Average degree	Average path distance	Nodes with max degree
SG_N_network	105	223	0.832	4.248	1.569	OTU_21939
SG_S_network	145	386	0.822	5.324	2.877	OTU_21939

### Disease-related and organic remediation-related bacteria enriched in swan geese’s guts in Poyang Lake

**Figure 5:**
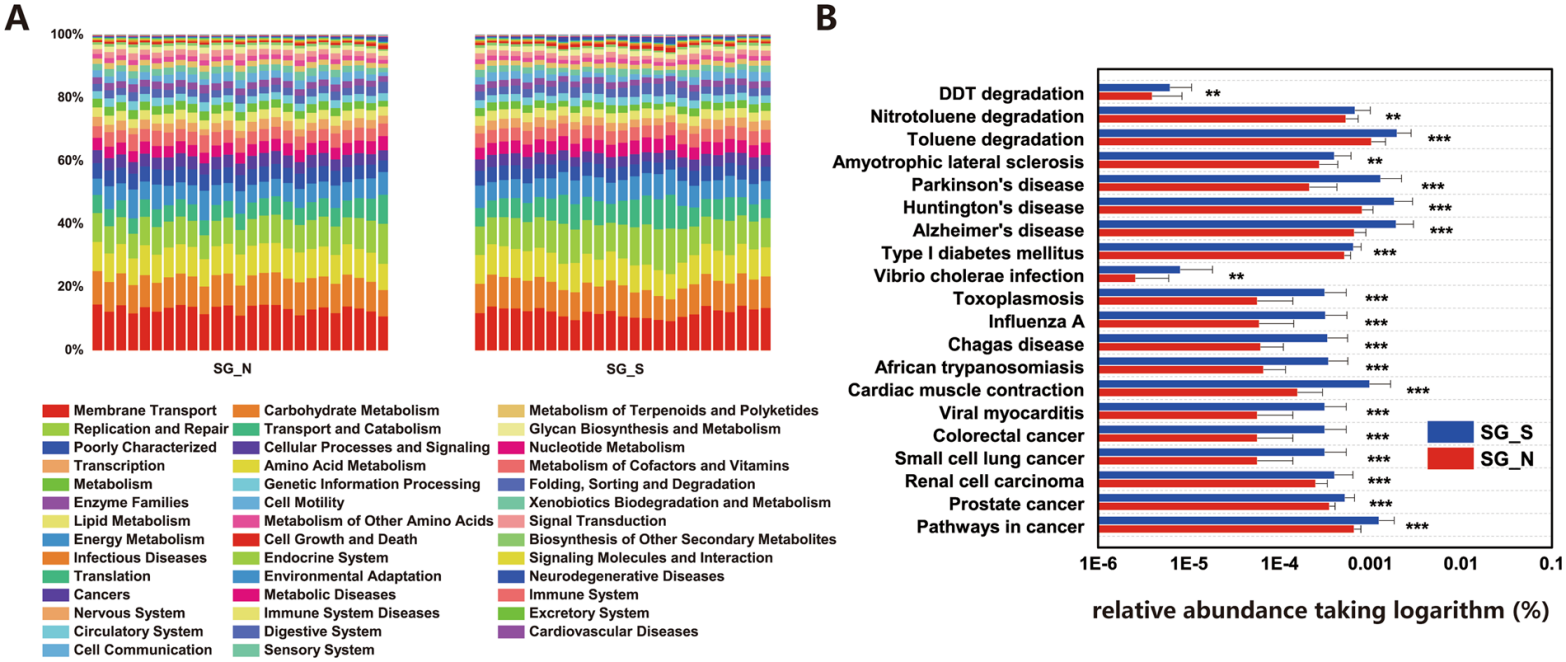
Functional predictions of all samples using PICRUSt. (**A**) Using PICRUSt as a predictive exploratory tool, comparing overall 41 level 2 KEGG Orthology groups (KOs) represented in data set between swan geese samples from two sites. (**B**) 3 xenobiotics biodegradation and 17 disease-related pathways between two sites. The x-axis represent relative abundance taking logarithm. **0.01 < *P* < 0.05, ***P < 0.01.

Using PICRUSt (Phylogenetic Investigation of Communities by Reconstruction of Unobserved States) as a predictive exploratory tool, we found that overall 41 of the 43 level II Orthology groups (KOs in KEGG (Kyoto Encyclopedia of Genes and Genomes)) were observed in the swan geese’s gut bacterial community (Fig. 5A). Almost half of the major functions were classified into multiple Metabolism groups (48.35%), including carbohydrate metabolism (9.60%), amino acid metabolism (9.19%), energy metabolism (6.27%), and glycan biosynthesis and metabolism (1.50%). Although the microbial composition was quite different among individuals (Fig. S5), their functional classification appeared to be more consistent (Fig. 5A) with most basic metabolic pathways being similar, however, several different pathways were still found (Fig. 5B).

Function prediction showed some differences in pathogenic and organic remediated functional taxa between swan goose gut microbiomes at two lakes (Fig. 5B). For pathogenic functions, samples from Poyang Lake consistently showed higher relative abundances than that of Khukh Lake, including ALS (Amyotrophic lateral sclerosis), Primary immunodeficiency, African trypanosomiasis, Toxoplasmosis, Colorectal cancer, Prion diseases, Bladder cancer, Vibrio cholerae infection, Measles, Phagosome, HCM (Hypertrophic cardiomyopathy), and Systemic lupus erythematosus (T-test, *P* < 0.005). Some organic biodegradation pathways also showed higher abundances in the southern habitat, including DDT degradation, nitrotoluene degradation, and toluene degradation (*P* < 0.05), indicating those related bacteria were enriched in the gut microbiome of swan geese at Poyang Lake.

### Species interaction networks between North and South habitats

**Figure 6:**
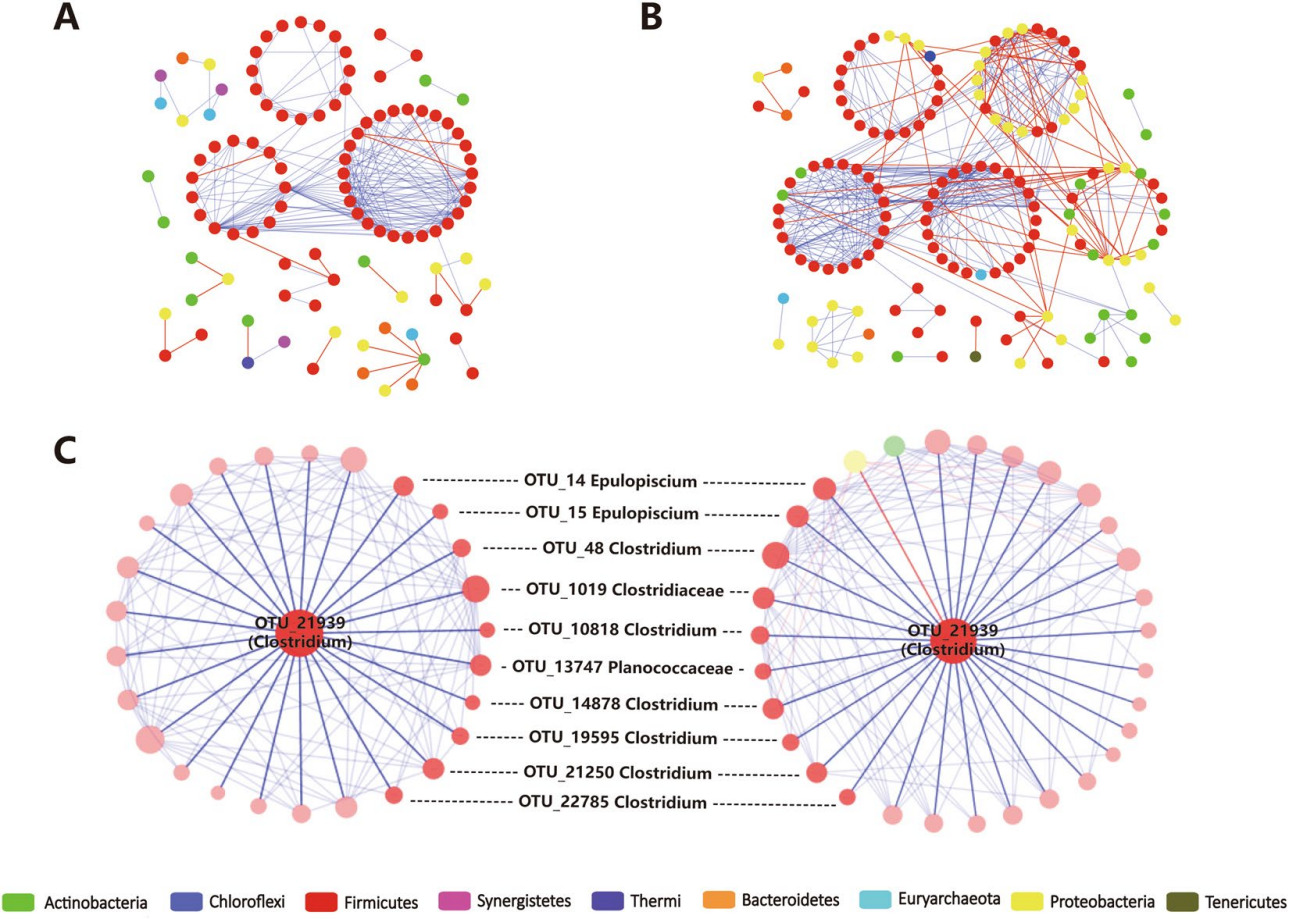
Phylogenetic Molecular ecology networks (pMENs) of swan geese bacterial species. (**A**) The pMEN of swan geese bacterial committee from Khukh Lake. (**B**) The pMEN of swan geese bacterial committee from Poyang Lake. (**C**) The nodes with max degrees in both (**A**) and (**B**) are identical. Its neighbors in (**A**) were plotted on the left and neighbors in (**B**) were on the right. The shared neighbors were linked with the dotted lines in the middle. The networks were constructed using RMT-based model and visualized by Cytoscape 3.3.0. Nodes represented OTUs, and lines connecting nodes (edges) represented positive (blue) and negative (red) interactions defined by Pearson’s correlation coefficient.

In order to understand species interactions in the gut microbiome after migration, pMENs (Phylogenetic molecular networks) derived from 16S rRNA data of swan geese were constructed. The co-occurrence patterns have been represented as links in a network which connect the nodes that represent different bacterial species. The South habitat gut microbiome pMEN was larger and more complex than the North habitat pMEN (Table 2, Fig. 6A,B), indicating microbiome at Poyang Lake showed more species interactions than gut microbiome at Khukh Lake. The different node typological positions in a network represented different roles for species in the community. Most of the nodes in these two sites are peripheral nodes while several module hub species were identified (Fig. S6). In these two pMENs, a majority of the nodes belonged to *Firmicutes*, the dominant phylum in swan geese gut microbiome, while the node with max degree (node with most neighbors) from both pMENs were the identical OTU_21939 which was an unknown species in genus *Clostridium*. Although 10 of its neighbors were consistent between both habitats (38% of neighbors in SG_N, 32% in SG_S), OTU_21939 still contained more distinctive neighbors in SG_N and SG_S (Fig. 6C), suggesting the interactions among the major species could be conserved to a certain extent but that most interactions could be swapped in these two areas.

## Discussion

Recent studies have explored the critical roles that animal gut microbiomes play in food digestion, metabolism regulation, and immune protection for wild animals^5,6^. Both host species and dietary nutrients are potential drivers of gut microbiome assemblies of wild animals^25,27^, while other factors could also alter gut microbiome composition, including age^30^, health^31^, and the habitat environment^22,24,32,33^. However, it is hard to distinguish its impacts with diet and host genetics in most cases. In this study, we attempted to track a group of swan geese via satellite and collected their fecal samples in different habitat areas separated by more than 1800 km. The breeding area, Khukh Lake, is located quite far from urban centers and is relatively undeveloped with low human population density (Fig. 1C). However, the wintering area located at Poyang Lake, the largest fresh water lake in China, has deteriorated in recent years because of industrialization and urbanization (Fig. 1D)^29,34^. Swan geese mostly eat the young stem of submerged macrophytes, particularly those of *Vallisneria spiralis*, in both wintering and breeding areas^2^. Therefore, these wild birds have a very narrow dietary range, such that the food sources are very similar in these two habitats. Due to the similar dietary regimes in both location, the influence of diet can be ignored as a factor, leaving local environmental factors as the main cause of microbiome differences. As a result, the fecal samples from these two sites showed some differences after the long migration. High-throughput sequencing of 16S rRNA and ITS from fecal DNAs were utilized to assess alterations to the gut microbiome composition, diversity, phylogenetic molecular networks, and functional predictions caused by long distance migration between breeding and wintering habitats.

First, we found that the core group of bacterial taxa in the swan goose gut microbiome are highly conserved after long-distance migration. Compared with the greater white-fronted geese from the same wintering habitat, the swan geese’s gut bacterial communities between showed less divergence between the two habitats (Fig. 4A and Table 1), suggesting the host species is still the most important factor in shaping the gut microbial community structure^25^. Additionally, Chao1 estimated richness and Shannon index showed that the alpha-diversity of swan goose bacterial communities between two sites showed no significant difference, and both of these values were lower than those of greater white-fronted geese (Fig. 3). At the phylum level, swan goose gut microbiome from both habitats was dominated by *Firmicutes*, which was in accordance with previous studies on other avian species, such as penguins^15^, *Procellariiform* seabirds^17^, chickens^35^, hooded cranes^18^, Swainson’s thrushes and gray catbirds^36^. Members of the phylum *Firmicutes* are associated with the decomposition of complex carbohydrates, polysaccharides, sugars and fatty acids, which are major nutrient sources for all animal hosts^37,38^. PICRUSt functional predictions also showed that metabolic pathways accounted the highest proportion (48.35%) of all classified bacterial functions (Fig. 5A). At the genus level, the dominant group for both sets of swan goose samples was *Clostridiaceae*-SMB53 (24.77% in SG_N and 21.58% in SG_S). In addition, the networking results showed that the central OTU (the node with most connectivity) was OTU_21939 (*Clostridium*) in the pMENSs from both sites. *Clostridium* was firstly discovered bacterial genus in human feces, and it is associated with production of acetic and formic acids^39^. All of the above results indicated the gut microbiome of swan geese, especially the dominant and central bacteria species, are consistent even after long-distance migration due to the host species and it’s restricted diet. A previous study of Swainson’s thrushes and gray catbirds also showed that energetic condition of migrants was not significantly related to overall microbiome community structure though it cannot be conclusively stated that migratory flight does not impact the microbiome^36^. Therefore, the core bacteria of the swan goose gut microbiome are conserved after migration.

However, several differences were found in terms of beta-diversity, composition, and interactions within swan goose gut microbiomes between breeding and wintering area. After long-distance migration, the phylogenetic beta-diversity of both bacterial and fungal communities showed significant changes, though it should also be noted there was a large amount of inter-individual variation (Fig. 4, Table 1). In the compositions of microbial communities, with some phyla and genera showing significant difference between two sampling sites. For example, *Proteobacteria* was the most obviously altered phylum between South and North habitats. Compared with the greater white-fronted geese from Poyang Lake, *Proteobacteria* in these two host species at the same site (Poyang Lake) showed no difference, but both of them were much higher in swan geese at Khukh Lake. *Proteobacteria* is the most dominant bacterial phylum in the freshwater systems of Poyang Lake^40^. Additionally, distinctive network topologies were found at each site (Table 2, Fig. 6). The more complex set of network interactions at Poyang Lake showed that microbial species interactions in geese’s gut could be altered according to host species^28^ and by habitat environmental stresses. Therefore, the gut microbiome of swan geese showed significant difference between the two habitats.

With the above results, we found that the general composition and central taxa of the wild swan goose gut microbiome were consistent between breeding and wintering areas due to the same bacterial and fungal species. However, significant divergences of both bacterial and fungal communities were detected between the two these habitats in terms of beta-diversity, community structures and microbial interactions due to the long-distance migration and differing environmental conditions at the two habitats. The breeding area was a lake that is minimally impacted by human activities, while the wintering area is highly impacted by human activities. The activities and behaviors of wild animals have always been affected by human activities^41^. Migratory animals have also been greatly threatened by human-induced change. For example, twenty years of continent-wide citizen science data assessing population trends of ten shorebird taxa that refuel on Yellow Sea tidal mudflats, a threatened ecosystem that has shrunk by > 65% in recent decades^24^. In recent years, the water quality of Poyang Lake has deteriorated sharply with the degree of eutrophication becoming more serious due to industrialization and urbanization, as well as a decrease in the size of the lake over time^29,42^. In this study, the gut microbiome of swan geese showed the obvious enrichment of pathogenic genera in Poyang Lake, including several *Gamma-proteobacteria* such as *Vibrio, Shewanella*, and *Pseudoalteromonas* (Fig. S3). In addition to those pathogenic genera, SG_S also had a large number of disease-related pathways than SG_N (Fig. 5B). Xenobiotics biodegradation and metabolism pathways, such as DDT degradation, Nitrotoluene degradation, and Toluene degradation, were also higher in swan geese’s gut microbiome in Poyang Lake than uncontaminated Khukh Lake. On the other hand, the network from Poyang Lake was more complex and showed a greater negative correlation, which may be related to environmental stress. Together these results help us to understand the impact of environmental pollution on wetlands, and especially its impact on the migratory birds that utilize those environments. It also supports efforts for the protection of these habitats for migratory birds, and provides basic data regarding the effects of habitat pollution and degradation on the birds.

In summary, the general composition and central taxa of the wild swan goose gut microbiome was generally unaffected by long-distance migration. However, significant divergences of both bacterial and fungal communities were also detected between the two typical habitats in terms of beta-diversity, community structures, and microbial interactions. Additionally, many disease-related and xenobiotics biodegradation pathways, pathogenic bacteria, and negative correlation were found in bacteria communities of samples in Poyang Lake, indicating the impact of habitat environment on the gut microbiome of wild animals. Our results revealed the variety of wild swan geese during their migration. We also indicated that, with the exceptions of food nutrients and host genetic distinctions, the habitat environments could alter the gut microbial communities of wild animals leading to the enrichment of some environmental-specific species in their intestines. Thus, polluted habitats may pose serious health threats to wild animals in part through alterations to their gut microbiome.

## Materials and Methods

### Sample collection

A flock of swan geese were satellite-tracked during migration from breeding to wintering grounds by fitting either 45 g or 70 g solar-powered Argos-GPS platform transmitter terminals to the backs of selected adults, using a teflon-ribbon harness (Fig. 1A). As swan geese are protected by regulations, their fresh feces were collected for DNA extraction without any direct hunting or injury during sampling. At the northern sampling site, fecal samples of swan geese were collected in Mongolia, Khukh Lake (N 49°28′07.79′′ E115°34′51.33′′) in August, 2014 (Fig. 1C). In South, feces were collected at Poyang Lake (N 28°54′20.22′′ E 116°16′13.92′′) in China, in January, 2015 (Fig. 1D). Poyang Lake National Nature Reserve is one of the most important wintering area for migratory geese in China, providing luxuriant food and suitable habitats for a high diversity of herbivorous wintering birds. Access into the lake areas were permitted by the Management Bureaus of Khukh Lake and Poyang Lake National Nature Reserve who are responsible for protection and management of these two lakes, respectively.

In this study, we collected 40 swan goose fecal samples from the two lakes, 20 fecal samples from Khukh Lake and 20 feces from Poyang Lake. Recent population counts indicate that a severe decline from 10,000–20,000 birds, to approximately 1,000 currently, has taken place during the last five years^1^. Based on recent waterbird surveys, we selected sites that hosted greater than 1% of the swan goose population, and waited while geese were feeding. As soon as the swan geese finished feeding and flown away, fresh droppings were collected and stored in sterile tubes. Each sample was collected with a minimum distance of two meters and was visually distinguishable among individuals. All samples were transported via dry ice and stored at −80 °C until further processing^43^.

### DNA extraction, PCR amplification and High-throughput sequencing

DNA extraction was carried out using a modified CTAB protocol^44^, with minor alteration in incubation time (to 12 h). Negative controls (extraction without feces) were included to monitor possible contamination for each batch of DNA extraction.

The extracted DNA was used as template for amplification of the V4 region of the 16S rRNA gene, which has high sequence coverage for prokaryotes and produces an appropriately sized amplicon, with a barcode primer set (515F, 5′-GTGCCAGCMGCCGCGGTAA-3′ and 806R, 5′-GGACTACHVGGGTWTCTAAT-3′)^45^. Meanwhile, we used a second barcode primer set (7F, 5′-GTGARTCATCGARTCTTTG-3′ and 4R, 5′-TCCTCCGCTTATTGATATGC-3′) targeting the ITS1 region of the rRNA operon of fungi for Illumina sequencing^46^. Both primers contained Illumina adapters and the reverse primer contained a 12 bp barcode sequence unique to each sample. The PCR amplification was carried out in a total reaction volume of 25 µl with three replicates for each sample. PCR amplification was under the following condition: initial denaturation at 94 °C for 1 min, followed by 30 cycles of 94 °C for 20 s, 57 °C for 25 s and 68 °C for 45 s, ending at 68 °C with a final extension step of 10 min.

All PCR amplifications were performed in triplicate and then combined. PCR amplicons were then pooled in equimolar concentrations on a 1% agarose gel, and purified PCR products were recovered using QIAquick gel extraction kit. High-throughput sequencing of the PCR products was performed on Illumina Miseq platform (Miseq PE250) at Central South University, Changsha, China.

### Sequence data processing

All raw 16S and ITS amplicon sequences were quality trimmed using Btrim^47^ and assigned to their respective samples according to the unique nucleotide barcodes. After removal of barcodes and primers, pair-ended sequences were merged and quality filtered by Flash^48^. Sequence data of the greater white-fronted geese fecal sample from Poyang Lake were downloaded from GenBank SRA with accession number of SRP078554 based on our previous study^28^. All previous sample collection, extraction and amplification steps were the same to swan geese samples. These sequences were then clustered into OTUs (operational taxonomic units) with a sequence threshold of 97% similarity by UPARSE^49^ and representative sequences of OTUs were picked up simultaneously. The singletons and chimeras were removed during the UPARSE procedure.

Taxonomic assignment of 16S representative sequences was carried out with the RDP (Ribosomal Database Project) classifier^50^ based on Greengene database^51^ and sequences (OTUs) assigned to Cyanobacteria were excluded from subsequent analysis^52^. Taxonomic assignment of ITS representative sequences was carried out with the RDP classifier based on the Warcup database^53^. Resampled 16S OTU subsets (15000 sequences per sample) and ITS OTU subsets (5000 sequences per sample) were used to calculate alpha diversity and beta-diversity. In this study, we calculated four kinds of a-diversity to measure the biodiversity of microbial community in geese gut. Richness was obtained by counting the observed species numbers associated with rarefaction curve. Chao1 value, Shannon indexes and Inverse Simpson indexes were calculated according to species abundance using vegan package (v.2.3–5) in R (v.3.2.5).

### Statistical analysis

All the representative sequences were aligned using PyNAST^54^. A phylogenetic tree was constructed with FastTree^55,56^. UniFrac was carried out with the phylogenetic tree to perform phylogenetic beta diversity analysis^57^. The differences in beta-diversity of bacterial communities were presented by PCoA. Student’s T test was employed to determine whether the distance between breeding and wintering habitats were significantly different using SPSS. PERMANOVA (Permutational multivariate analysis of variances) test and ANOSIM (Analysis of Similarity) were carried out to test whether the gut microbiome structure was significantly different between two sites using method implemented in the R package vegan^58^. LefSE was utilized to show the comparison of swan geese bacterial species between the two habitats at all levels^59^.

### Functional predictions using PICRTUSt

PICRUSt is a bioinformatics tool that uses marker genes to predict gene family abundance in environmental DNA samples for which only marker gene data are available^60^. In this study, we employed this method to predict the molecular functions of each sample based on 16S rRNA sequencing data. We used the KEGG database and performed closed reference OTU picking using the sampled reads against a Greengenes reference taxonomy (Greengenes 13.5). The 16S copy number was then normalized. After that, molecular functions were predicted and final data were summarized into KEGG pathways.

### Network construction

The correlation network was constructed from OTUs as previously described based on RMT (Random matrix theory)^61^. RMT-based approach is a way to construct pMENs which will represent various biological interactions in gut microbial communities^62^. By determining the most interacted microbial taxa, networks can also identify the keystone species, which might have largest effect on microbial community structure and potential functions^63^. In this study, molecular networks were constructed using RMT models after data standardization and Pearson correlation estimation^62,64^. In order to characterize the modularity property, each network was split into modules. Zi indicates how well a node connects to nodes within the same module, while Pi indicates how well a node connects to other modules. Based on within-module and among-module connectivity, topological roles of different nodes were divided into four categories, (i) network hubs: nodes with Zi > 2.5 and Pi > 0.6; (ii) module hubs: nodes with Zi > 2.5 and Pi ≤ 0.6; (iii) connectors: nodes with Zi ≤ 2.5 and Pi > 0.6; and (iv) peripheral nodes: nodes with Zi ≤ 2.5 and Pi ≤ 0.6^65^. We then analyzed the global network properties and individual node’s centrality, separated each module and calculated modularity using Cytoscape 3.3.0^66^. PMENs aimed to show whether the gut microbiome tended to be more closely related in case of environmental stress.

### Ethical approval

During this study, we fitted 45 g or 70 g solar-powered Argos-GPS platform transmitter terminals to the backs of selected adults, using a teflon-ribbon harness. In this way, neither will the transmitters harm their bodies, nor will they increase the burden of birds’ flying. Their fresh feces were collected for DNA extraction without any direct hunting and injury involved during the sampling collection. No direct capture or hunting involved. Lake access was permitted by Wetland National Nature Reserve Administration, agencies which are responsible for the protection and management of these two lakes.

### Accession codes

Raw sequences were submitted to the NCBI Sequence Read Archive (SRA) under the accession number SRP094679.

## Conclusion

In summary, the general composition and central taxa of the wild swan goose gut microbiome were relatively unchanged after long-distance host migration. However, significant divergences of both bacterial and fungal communities were also detected between the two typical habitats in terms of beta-diversity, community structures and microbial interactions. Additionally, many disease-related and xenobiotics biodegradation pathways, pathogenic bacteria, and negative correlation were found in bacteria communities of samples in Poyang Lake, indicating the impact of habitat environment on the gut microbiome of wild animals. Our results revealed that excepting for food nutrients and host genetic distinctions, the habitat environment could also alter the gut microbial communities of wild animals and enrich some environmental-specific species in their intestines. Thus, the polluted habitats are serious health threats to all wild animals in part through their gut microbiomes.

## Electronic supplementary material

Supplementary Information
