# Supplementary material for: Habitat environments impacted the gut microbiome of long-distance migratory swan geese but central species conserved
Source: Sci Rep. 2018 Sep 6;8:13314. doi: 10.1038/s41598-018-31731-9 (PMC6127342; doi:10.1038/s41598-018-31731-9)

Supplementary Information **for** ***Scientific Reports***

Habitat environments impacted the gut microbiome of long-distance migratory swan geese but central species conserved

Yueni Wu[1,2], Yuzhan Yang[1], Lei Cao[2,3], Huaqun Yin[4], Meiying Xu[5], Zhujun Wang[1,2], Yangying Liu[1,2], Xin Wang[2,3], Ye Deng[1,2]

[1] CAS Key Laboratory for Environmental Biotechnology, Research Center for Eco-Environmental Sciences, Chinese Academy of Sciences, Beijing, China

[2] College of Resources and Environment, University of Chinese Academy of Sciences, Beijing, China

[3] State Key Laboratory of Urban and Regional Ecology, Research Center for Eco-Environmental Sciences, Chinese Academy of Sciences, Beijing, China

[4] School of Minerals Processing and Bioengineering, Central South University, Changsha China

[5] State Key Laboratory of Applied Microbiology Southern China, Guangdong Institute of Microbiology, Guangzhou, China.

Correspondence

Ye Deng

18 Shuangqing Road, Haidian District, Beijing 100085, China P.R.

Tel: 00 86 (010) 6284 0082

Fax: 00 86 (010) 6284 0082

yedeng@rcees.ac.cn

Supplementary Figure S1: Rarefaction Curve of 16S and ITS sequencing.

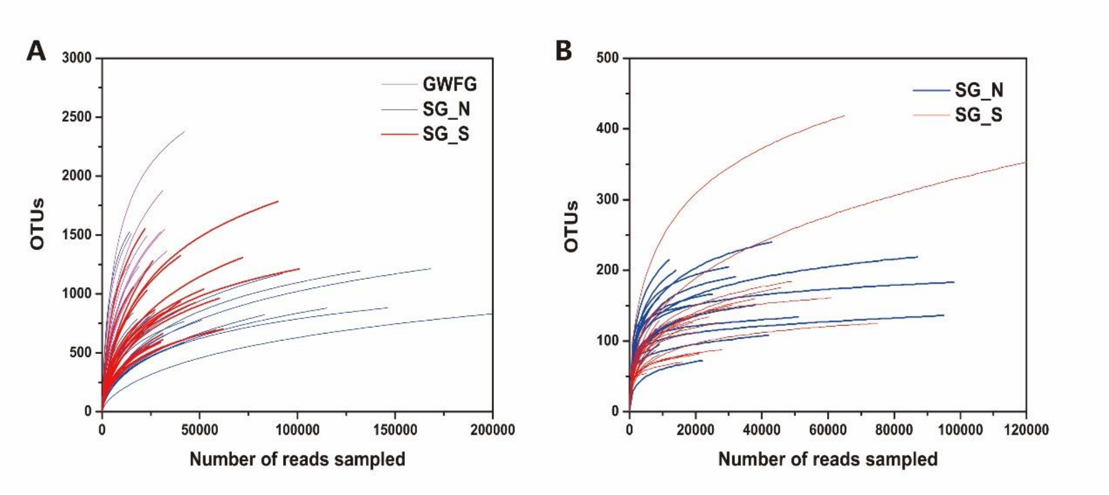

Supplementary Figure S2: Phylum level gut microbiome composition of swan geese of three dominant bacteria phylum.

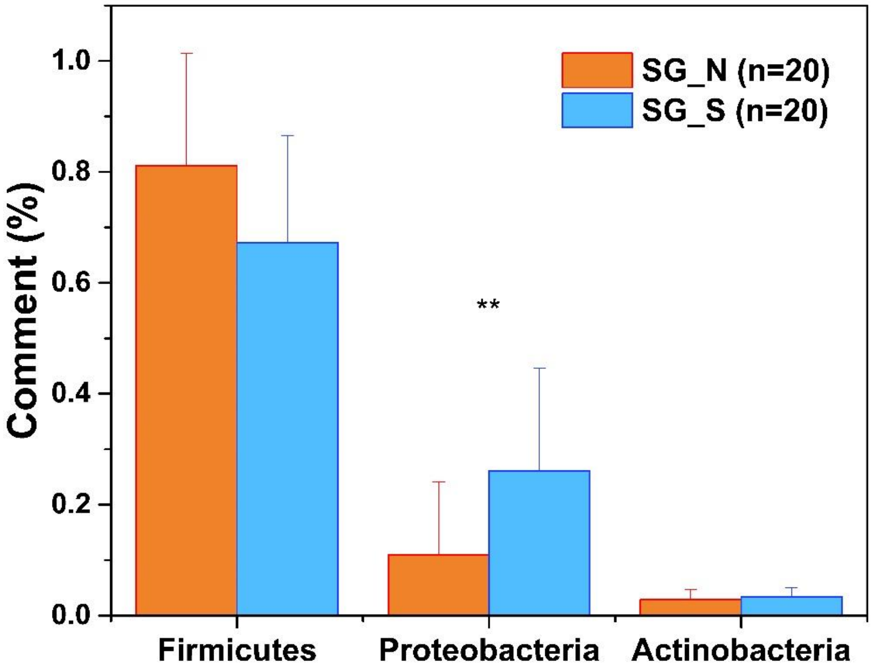

Supplementary Figure S3: Heatmap showing the relative abundance of some selected species across herbivorous geese.

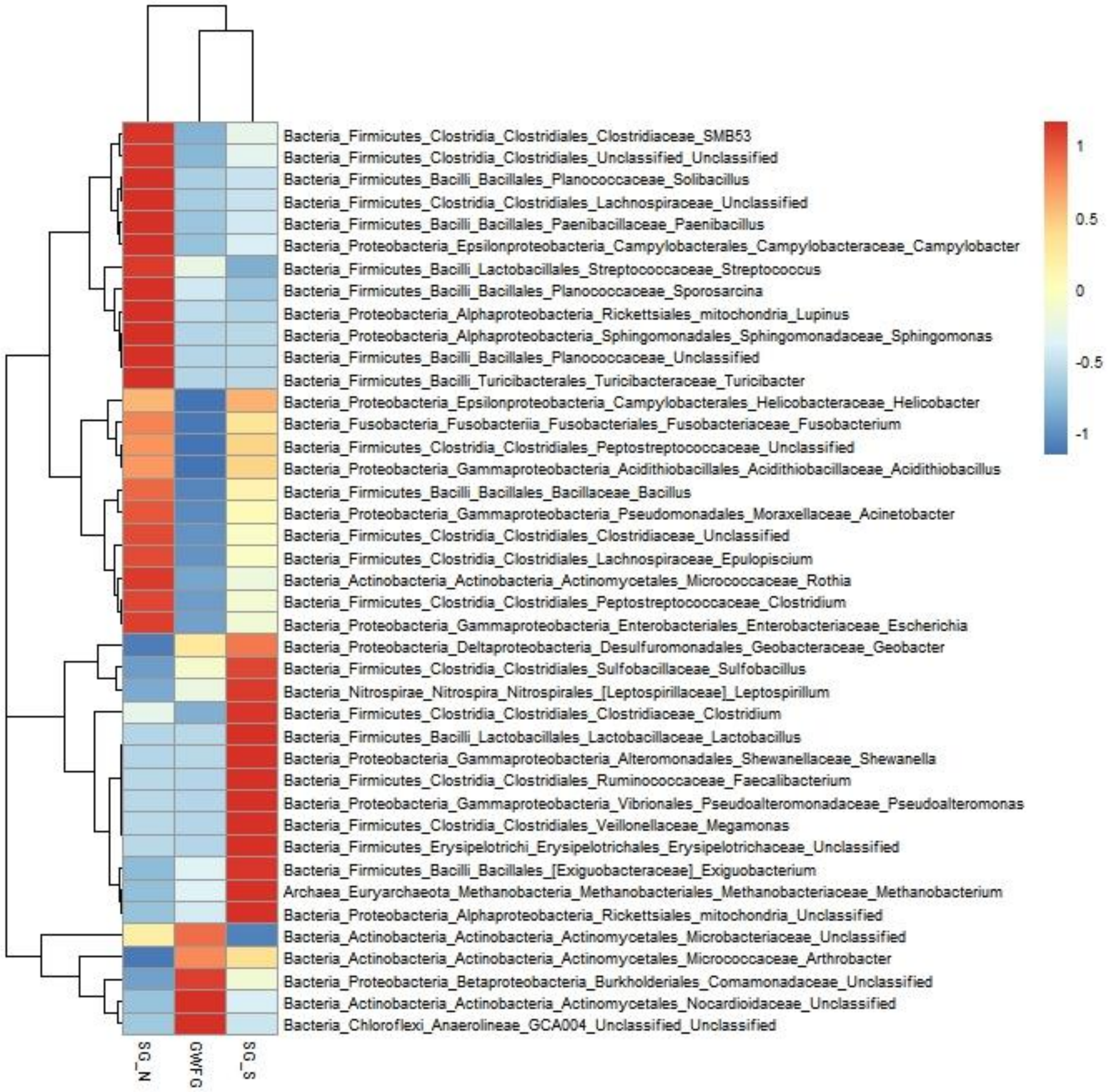

Supplementary Figure S4: LefSe showing comparison of swan geese bacterial species between two habitats at all levels. (A) The module plots the biomarkers found by LEfSe ranking them accordingly to their effect size and associating them with the class with the highest median. (B) The raw data of a single feature as an abundance histogram with four significant different dominant genus. (C) This module produces cladograms representing the LEfSe results on the hierarchy induced by the label names. Red blocks represent significant difference at genus level. Blue blocks represent significant difference of *Proteobacteria*.

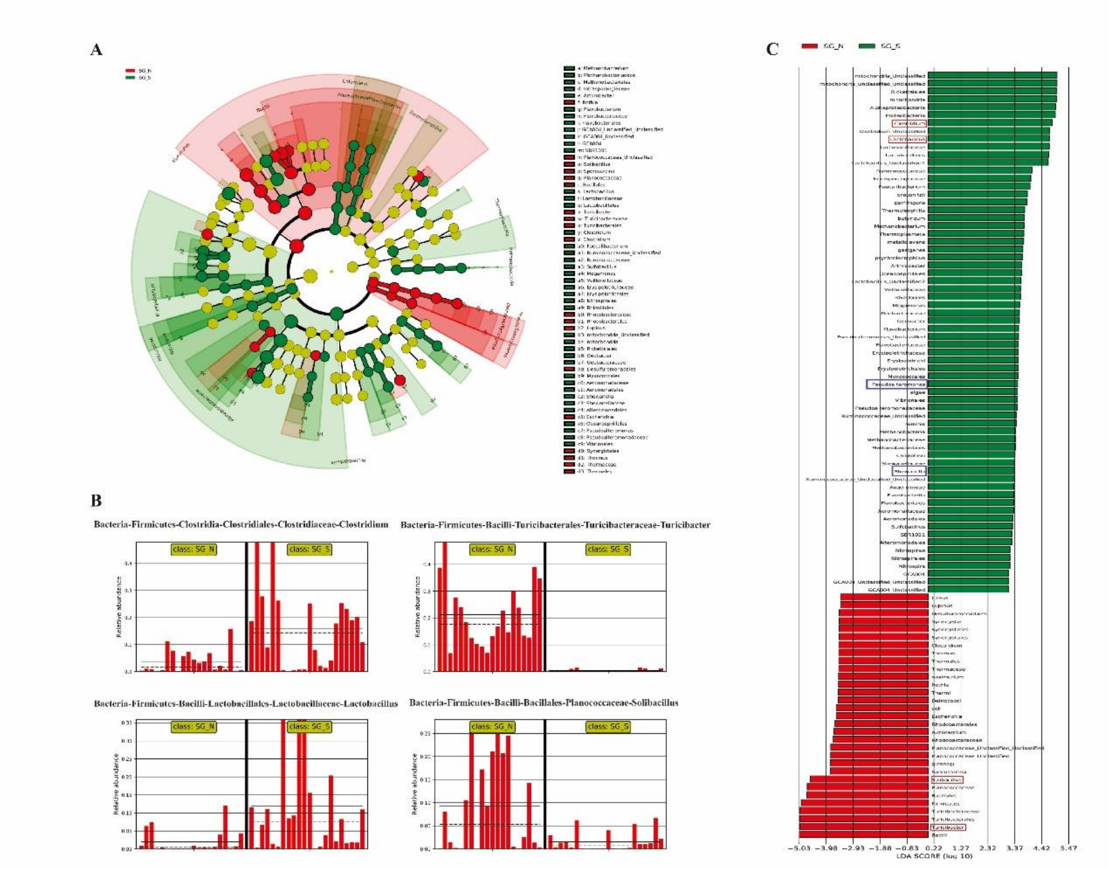

Supplementary Figure S5: Phylum level individual gut microbiome composition of swan geese including bacteria and fungi community.

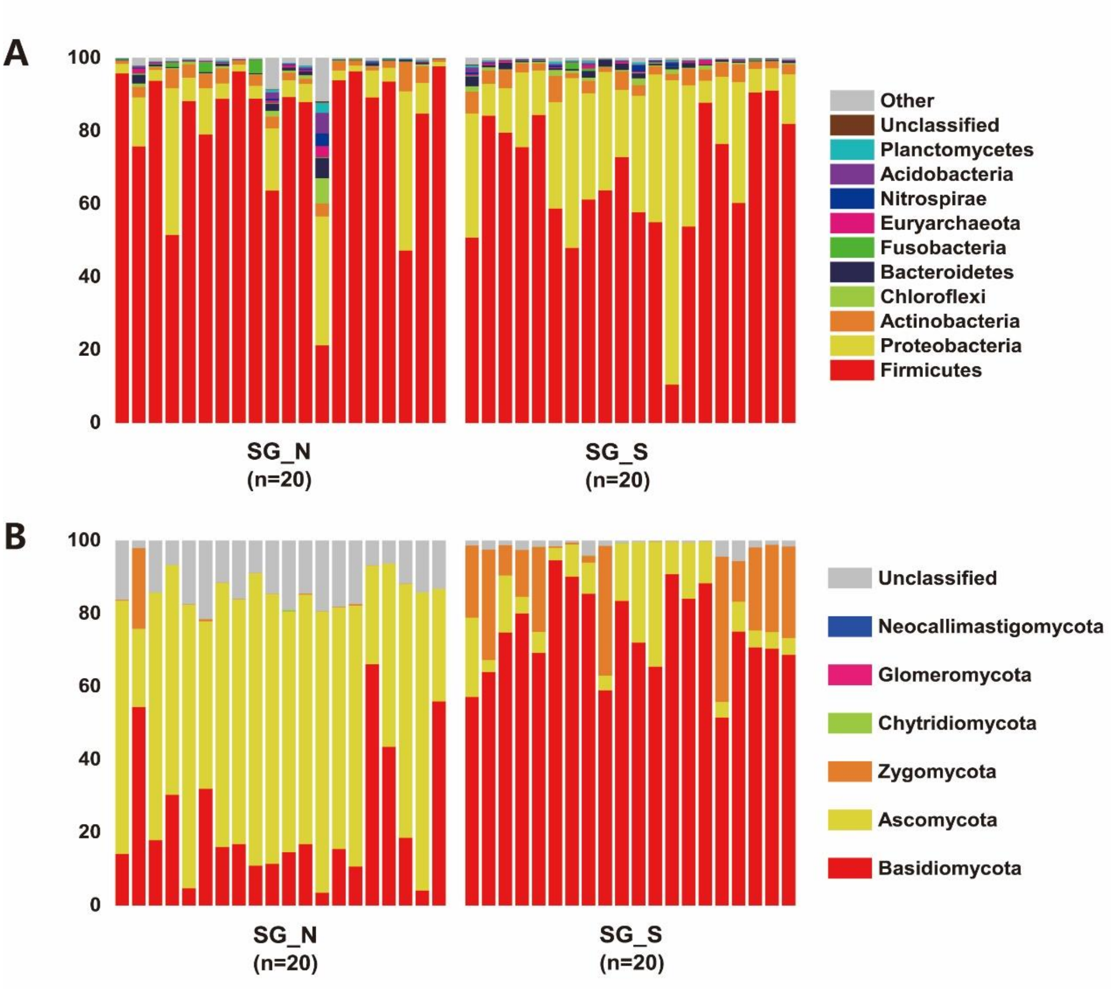

Supplementary Figure S6: Z-P plot showing the distribution of OTUs based on their topological roles.

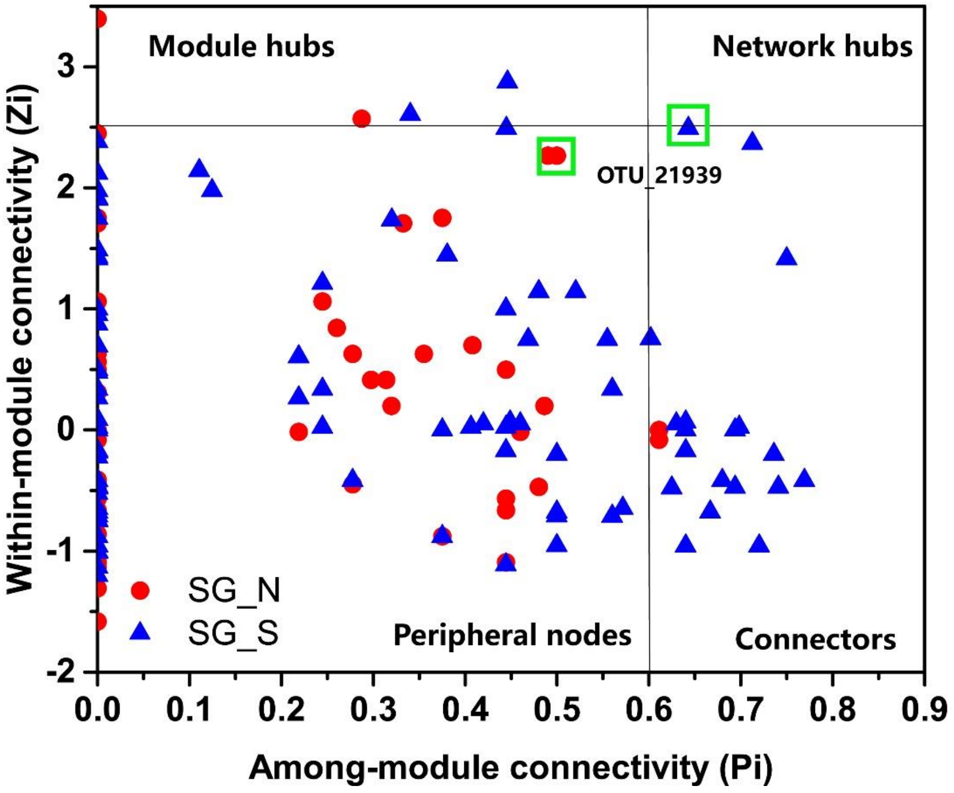

Supplement: Supplementary file 1 — Supplementary Information [file 41598_2018_31731_MOESM1_ESM.pdf]
